# Murine Myoblasts Exposed to SYUIQ-5 Acquire Senescence Phenotype and Differentiate into Sarcopenic-Like Myotubes, an In Vitro Study

**DOI:** 10.1093/geroni/igaf122.2053

**Published:** 2025-12-31

**Authors:** Laura Gerosa, Giovanni Lombardi, Guiseppe Banfi, Amir Malvandi, Marta Gomarasca, Chiara Verdelli, Veronica Sansoni, Martina Faraldi

**Affiliations:** IRCCS Ospedale Galeazzi, Milan, Italy; IRCCS Ospedale Galeazzi - Sant'Ambrogio, Milan, Italy; IRCCS Ospedale Galeazzi - Sant'Ambrogio, Milan, Italy; IRCCS Ospedale Galeazzi - Sant'Ambrogio, Milan, Italy; IRCCS Ospedale Galeazzi - Sant'Ambrogio, Milan, Italy; IRCCS Ospedale Galeazzi - Sant'Ambrogio, Milan, Italy; IRCCS Ospedale Galeazzi - Sant'Ambrogio, Milan, Italy; IRCCS Ospedale Galeazzi - Sant'Ambrogio, Milan, Italy

## Abstract

Aging profoundly affects the musculoskeletal system, where bone and skeletal muscle function as an integrated unit. The accumulation of senescent cells contributes to functional decline in both tissues, but the underlying molecular mechanisms remain poorly understood, partly due to the lack of reliable in vitro models. To address this, we developed a novel in vitro model of muscle cell senescence using mouse C2C12 myoblasts that, when subjected to differentiation, the resulting myotubes showed sarcopenic features. Senescence was induced by SYUIQ-5, a quindoline derivative that inhibits telomerase activity and triggers a senescent phenotype characterized by cell cycle arrest, increased p21 expression, enhanced senescence-associated β-galactosidase (SA-β-gal) activity, and phosphorylation of p53 and histone H2AX. These senescent cultures displayed impaired differentiation capacity, forming myotubes with sarcopenic features including elevated ubiquitin ligases, ATROGIN-1 and MURF1, reduced mitochondrial content, and increased secretion of myostatin, a negative regulator of muscle growth, suggesting that a greater burden of senescent muscle cells could contribute to sarcopenia. This developed model enables the investigation of the dynamic crosstalk between muscle and bone during aging, shedding light on the pathological mechanisms that jointly contribute to the deterioration of both tissues specifically exploring the role of muscle cell senescence. In this framework, we employed untargeted metabolomic and lipidomic profiling to examine metabolic alterations in senescent muscle cells and examined the contribution of the senescence-associated secretory phenotype (SASP) and extracellular vesicles (EVs), and their miRNA content, as mediators of altered inter-tissue communication. Overall, these findings present a robust and well-characterized in vitro model of muscle cell senescence, offering a powerful platform for uncovering the molecular drivers of musculoskeletal aging. Indeed, the first studies conducted using this system are already uncovering potential molecular pathways linking muscle senescence to bone dysfunction, paving the way for the identification of novel biomarkers and therapeutic targets associated with sarcopenia, frailty, and age-related musculoskeletal disorders.

